# Senescence-related gene signature predicts prostate cancer progression and identifies PCNA as a therapeutic target via multi-omics machine learning integration

**DOI:** 10.1038/s41416-025-03309-6

**Published:** 2025-12-18

**Authors:** Renxuan Lin, Hiocheng Un, Youmei Kang, Jiahao Lei, Lingwu Chen, Ren Liu, Zongren Wang

**Affiliations:** 1 Department of Urology, First Affiliated Hospital of Sun Yat-sen University, Guangzhou, China; 2 Institute of Precision Medicine, First Affiliated Hospital of Sun Yat-sen University, Guangzhou, China

**Keywords:** Tumour biomarkers, Targeted therapies

## Abstract

**Background:**

Senescence plays a critical role in prostate cancer, influencing disease onset and progression. However, the alterations of senescence-associated genes during prostate cancer progression and their potential value in predicting disease advancement remain to be further elucidated.

**Methods:**

117 machine learning methods were applied to construct the senescence-related gene signature (SRGS). Temporal trajectory analysis based on bulk and single-cell transcriptomic datasets was performed to link SRGS with prostate cancer progression. Functional validations of PCNA were conducted both in vitro and in vivo to support our analytical findings.

**Results:**

Using 117 machine learning methods, we developed the SRGS, which demonstrated robust predictive capability across multiple cohorts, including our own cohort of 90 patients. The SRGS also showed strong potential in predicting overall survival in patients treated with second-generation AR inhibitors. Temporal trajectory analysis of bulk RNA-seq and single-cell data revealed the biological significance of SRGS and identified Proliferating Cell Nuclear Antigen (PCNA) as a potential driver of PCa progression. Pharmacological inhibition of PCNA with AOH1996 significantly suppressed tumor growth and enhanced the efficacy of androgen deprivation therapy.

**Conclusion:**

We developed the SRGS that effectively predicts prostate cancer prognosis and progression. Moreover, our findings highlight PCNA as a promising therapeutic target in PCa.

**Figure:**
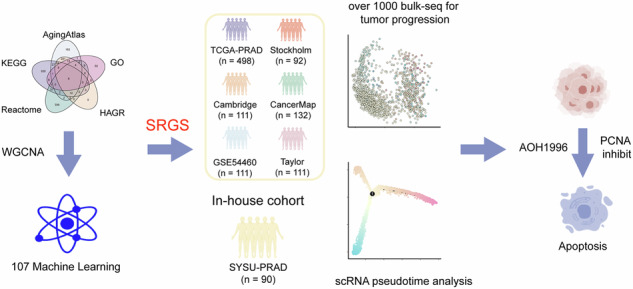
Integrated analysis of multi-cohort transcriptomic data developed an SRGS enabling accurate prognostication and identification of high-risk patients. Results highlight SRGS’s clinical utility and nominate PCNA as a promising therapeutic target in high-risk and castration-resistant prostate cancer (CRPC).

## Introduction

Prostate cancer (PCa) remains a major contributor to cancer-related mortality among men globally [1], with ~2.9 million new cases projected by 2040 due to an aging global population [2]. While genetic predisposition and family history contribute to PCa risk, advancing age is a primary driver, strongly associated with both increased incidence and more aggressive disease phenotypes [3, 4]. Aging is also a significant risk factor for various other diseases, as it encompasses cellular senescence, genomic instability, and disrupted tissue homeostasis [5].

Recent studies have revealed a strong association between senescence phenotypes and PCa progression [6, 7]. In the context of tumor progression, senescence is believed to suppress tumor growth, potentially explaining the relatively slow development of PCa [8, 9]. However, a subset of patients experience biochemical recurrence (BCR), which refers to disease relapse following radical prostatectomy, and this is often followed by rapid progression to lethal metastases [10]. Although current risk stratification including clinical parameters are used to estimate the likelihood of BCR in PCa, several studies have demonstrated that molecular biomarkers, such as Decipher, GPS, and Prolaris tests, exhibit superior predictive performance compared with clinical parameter [11–13]. Moreover, early identification of BCR risk and timely therapeutic intervention have been recognized as effective strategies to improve patient outcomes [14]. Androgen deprivation therapy (ADT) is generally effective as a first-line treatment for PCa following BCR. However, in some patients, the disease progresses to the castration-resistant prostate cancer (CRPC) stage, where it no longer responds to conventional ADT [15]. This transition is associated with a poor prognosis and remains a major challenge in PCa management. Previous studies have demonstrated that the primary cellular response to ADT in PCa may be senescence-associated growth arrest [16, 17]. However, emerging evidence shows that senescent cells can regain proliferative capacity, contributing to disease recurrence and progression [18].

Therefore, from both progression and resistance perspectives, there is a critical need for reliable clinical prognostic models to guide precision medicine. Developing predictive models based on senescence-related gene signatures to assess disease progression and treatment resistance, along with strategies to prevent the reactivation of proliferative potential in senescent cells, may represent a promising therapeutic approach to prevent or delay the progression of CRPC.

To decode the dynamic role of senescence in PCa progression, we integrated transcriptomic data from six independent cohorts and applied 117 machine learning methods to construct a robust senescence-related gene signature (SRGS) predictive of BCR. The predictive power of the SRGS was further validated in our own sequencing cohort. In addition, temporal trajectory analysis of bulk and single-cell RNA-seq data confirmed the association of SRGS with disease progression and identified Proliferating Cell Nuclear Antigen (PCNA) as a potential driver of the tumor progression. We further validated through in vitro and in vivo experiments that pharmacological inhibition of PCNA may block this transition, offering a novel therapeutic strategy for high-risk PCa.

## Materials and methods

### Data processing

PCa transcriptomic datasets with BCR-free survival information were obtained from six publicly available sources: TCGA-PRAD, Stockholm, Cambridge, CancerMap, GSE54460, and Taylor (retrieved on January 12, 2023), along with corresponding clinical annotations (see Supplementary Table 1). Inclusion criteria required: (1) primary PCa tissue samples; (2) available RNA-seq data; (3) documented radical prostatectomy (RP); and (4) at least 30 days of postoperative follow-up. The normal prostate tissue data from GTEx were obtained via https://xenabrowser.net/.

Our study did not involve any clinical research. For our in-house cohort (SYSU-PRAD cohort), informed consent was obtained from all participants. The study was approved by the Research Ethics Committee of the First Affiliated Hospital of Sun Yat-sen University ([2022] No. 101). Fresh radical prostatectomy specimens were collected from patients with primary PCa. Samples with available bulk RNA sequencing, whole-exome sequencing (WES), and proteomic data were included to constitute our in-house cohort. The transcriptomic, proteomic, and WES sequencing were performed by Shanghai Zhongke New Life Biotechnology Co., Ltd. Single-cell RNA sequencing (scRNA-seq) was conducted on the 10x Genomics platform by Lianchuan Biotechnology Co., Ltd.

Normalization strategies were applied according to data type: TCGA and SYSU-PRAD RNA-seq data were converted to FPKM values; SRA datasets were normalized using the trimmed mean of *M*-values (TMM) method through the edgeR package; and microarray data from CancerMap and Taylor were normalized using robust multi-array average (RMA) through the oligo package. Preprocessed expression data from cBioPortal and GEO was used without additional processing. All expression data were log2-transformed to ensure uniformity prior to downstream analyses.

### Collection of senescence-related genesets

Human senescence-related genesets were obtained from five manually curated and publicly available resources, namely AgingAtlas [19], KEGG [20], and Reactome [21], Human Ageing Genomic Resources (HAGR) [22] and GO [23], by retrieving keyword “senescence”.

### Weighted correlation network analysis (WGCNA)

The WGCNA package was used for generating co-expression networks of TCGA-PRAD [24], with an optimal soft threshold (β) determined to ensure compliance with the scale-free network criteria. In addition, the weighted adjacency matrix was transformed into a topological overlap matrix (TOM) before computing the corresponding dissimilarity (1TOM). Gene modules were then identified through the dynamic tree cutting approach, with the red module exhibiting the most significant correlations with clinical index. Genes with both high GS and MM were used for further study.

### Construction of SRGS model

A consensus SRGS with high levels of stability and accuracy was developed by integrating 10 machine learning algorithms using Mime package. These included partial least squares regression for Cox (plsRcox), survival support vector machine (Survival-SVM), generalized boosted regression modeling (GBM), random survival forest (RSF), least absolute shrinkage and selection operator (LASSO), stepwise Cox, Ridge, CoxBoost, elastic network (Enet), and supervised principal components (SuperPC) for model construction. Additionally, Harrell’s concordance index (C-index) was determined in five datasets for validation, with the model having the highest average C-index subsequently selected.

### Bulk pseudo-time analysis of prostate cancer disease progression

Human RNA-Seq datasets were obtained from Bolis et al. [25]. We calculated the pseudotime score for each sample using the ProstateCancerAtlas (https://prostatecanceratlas.org/app/home). The pseudotime trajectory was derived by applying slingshot, with principal components (PC1 and PC2) from PCA as input, assigning each sample a pseudotime value ranging from 0 to 250. GSEA analysis was used to quantify GSEA score for nine genes which constructing SRGS. Pearson’s correlation analysis was used to analyze the GSVA scores and disease progression (pseudotime). The detailed computational methodology can be referred to in the previously published study by Bolis et al. [25].

### Single cell RNA-seq

Single cell transcriptomic data were acquired from our own single-cell datasets comprising 5 primary PCa samples and GSE137829 comprising 5 CRPC samples [26]. Cells were discarded if they expressed more than 6000 genes, less than 500 genes or less than 300 UMI counts. Those where the ratio of mitochondrial expression to endogenous gene expression exceeded 15% were also discarded. Using the NormalizeData function, a log_2_ transformation was then applied to the raw data for normalization prior to scaling with the ScaleData function. Additionally, the FindVariableFeatures function (selection.method = “vst”) was used for computing the top 2000 genes having the highest standardized variance, with principal component analysis (PCA) also conducted using the default parameters in the RunPCA function. We performed batch correction by R package Harmony (version 0.1.0). Putative doublets were identified and discarded using scDblFinder R package (v 1.4.0). Potential doublets, identified based on the coexpression of different well-known cell-type markers, were filtered out, leaving 50992 cells with identified subsets. The SRGS score of single-cell was calculated by AddModuleScore. The infercnv package in R (v 1.14.2) was utilized to infer single-cell copy number variants. The CNV score for each cell was calculated using ∑(CNVᵢ − 1)², where CNVᵢ represents the inferred CNV level for region i.

### Differential expression and enrichment analyses

Differentially expressed genes were computed using FindAllMarkers functions (test.use = “wilcox”, min.pct = 0.25). In this case, genes were considered as being differentially expressed using an absolute log2(fold change) of >0.25 and an adjusted *P* value of <0.05 as threshold. For enrichment analysis, KEGG terms were identified using enrichCluster, with results considered to be statistically significant at a P-adjusted value of <0.05, following Benjamini–Hochberg (BH) correction.

### Single-cell trajectories

We utilized Monocle (v 2.24.1) to order epithelial cells in pseudotime based on their transcriptomic similarity. Cell states, groups and gene expression in pseudotime were plotted with plot_cell_trajectory.

### Cell proliferation assay

To each well of a 96-well plate, 2000 PC3 or DU145 cells were added. After treatment with a small molecule PCNA inhibitor AOH1996 (Abmole, M40519, China) for 24 h, CCK-8 solution (Abmole, M4839, China) was introduced to the wells for 2 h, and absorbances at 450 nm were read to assess cell viability.

The colony formation assay involved adding 1000 PC3 or DU145 cells to each well of 6-well plates, and after adhesion, a 24-h treatment was performed using AOH1996. After ~7 days of incubation, cell colonies were washed with 1x PBS prior to a 20-min staining with crystal violet. The colonies were subsequently imaged and quantified to assess colony formation ability.

Apoptosis Assay To each well of six-well plates, 2 × 10^5^ PC3 or DU145 cells were added along with 2 mL of complete medium. The next day, cells in the combined pan-apoptosis inhibitor and PCNA inhibitor group were pretreated with 40 µM V-ZAD-FMK (MCE, HY-16658B, China) for 30 min. Following pretreatment, 500 nM AOH1996 was added to both the pan-apoptosis inhibitor group and the PCNA inhibitor group, after which a 24-h incubation was performed. The recommended protocol of an Annexin V-FITC/PI staining kit (Biosharp, BL110A, China) was then followed to assess apoptosis levels. Fluorescence signals were detected by flow cytometry before quantifying the proportion of apoptotic cells.

### Immunohistochemistry

Orthotopic tumor tissues were collected from mice for immunohistochemistry staining. After fixing the tumor samples for 12 h in 4% paraformaldehyde (PFA), they were paraffin-embedded and sectioned (4 μm). The sections were boiled in Tris-EDTA for antigen retrieval, after which, 3% H2O2 was added to block peroxidases and nonspecific binding of antibodies. Additional blocking was then performed using 20% goat serum before overnight treatment (4 °C) with the primary antibody. The sections were then washed, and after a 30-min incubation with the secondary antibodies, they were stained with DAB (Aglient, K5007), followed by hematoxylin staining. A KF-PRO-020 Digital Slide Scanner (KFBio, China) was eventually used to scan the slides.

### TUNEL assay

The TUNEL assay was carried out as instructed in the 1-step TUNEL in situ apoptosis kit (Elabscience, E-CK-A325, China). Briefly, after deparaffinization of the slides, permeabilization was performed with Proteinase K solution for 20 min at 37 °C. The slides were then equilibrated with TdT Equilibration Buffer at 37 °C for 20 min. Next, the labeling solution was applied and incubated at 37 °C for 2 h. Finally, the cells were stained with DAPI solution for 5 min at room temperature. The result of cells showing TUNEL positivity was then examined and recorded using a fluorescence microscope.

### Animal models

All animal experiments complied with the ethics of animal experiments in the Experimental Animal Center of Sun Yat-sen University ([2025] no. 011). In each mouse model, five mice were randomly assigned to each group, and outcome measurements were performed without blinding. The Animal Experiment Center of Sun Yat-sen University provided 4-week-old C57BL/6 male mice for orthotopic injection. After anesthesia and lower abdominal incision, we exposed prostate to inject 1 × 10^6^ RM1 cell, *n* = 5 per group. Then we use 6–0 silk sutures to close the abdominal wall and skin. Enzalutamide (20 mg/kg, MCE, HY-70002, China) was orally administered five times per week for 2 weeks. AOH1996 (40 mg/kg, Abmole, M40519, China) was given daily through the oral route for 2 weeks. The animals were euthanized via rapid cervical dislocation and subsequent decapitation, with the tumor tissues rapidly extracted for analysis. For subcutaneous injections, 4-week-old male nude mice were inoculated with 5 × 10^6^ PC3 cells, *n* = 5 per group. Calipers were used to measure the length and width of tumors in two dimensions before calculating the tumor volume as follows: 0.5 × (length × width^2^).

### Statistics

Clinical data were analyzed using standard statistical tests, including the Log-rank test, Kruskal-Wallis test, Wilcoxon test, and Student’s *t* test. Spearman’s correlation was also used to analyze variables with a continuous distribution. All statistical tests were two-sided, with *P* < 0.05 indicating significance. To account for multiple-testing, the *P* value was adjusted based on the Benjamini-Hochberg FDR method. We used the survminer package to determine the optimal cut-off point for each cohort. The cohort-specific optimal cut-off point stratified patients into high- and low-risk groups. BCR-free survival was described using Kaplan–Meier plots (Log-rank test). The same analytical approach was also applied to evaluate the prognostic value of individual proteins. R (version 4.2.2) was used to analyze all clinical data, presented as the mean and standard deviation (SD) of at least three independently-replicated experiments, while GraphPad Prism (version 9.0.0) was utilized for statistical analyses.

## Result

### Identification of senescence-related genes associated with PCa prognosis

**Fig. 1:**
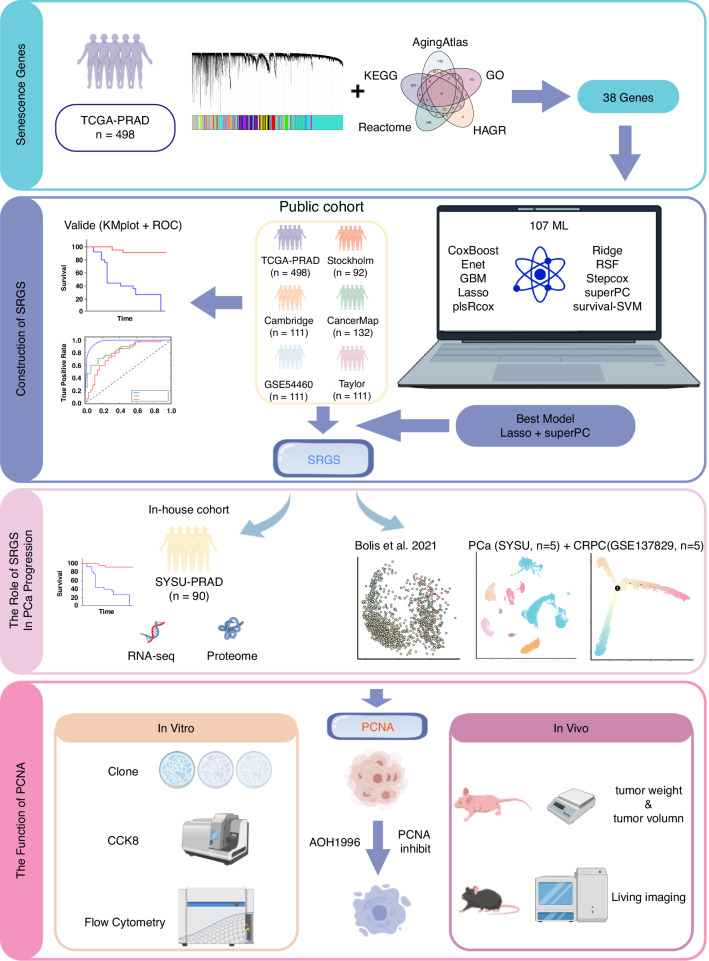
Illustration of the study design. The outline of the figure was obtained from the free website BioGDP.com (https://biogdp.com/).

**Fig. 2:**
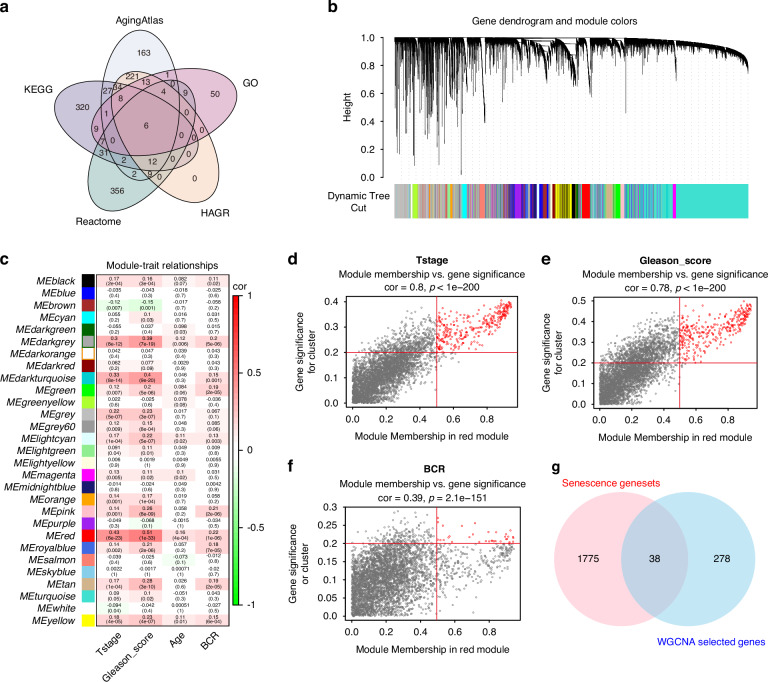
Identification of prostate cancer prognosis-associated senescence marker genes. **a** Senescence-related genes were collected from five databases. **b** Module genes were constructed using bulk RNA-sequencing data from TCGA-PRAD. **c** Correlation analysis was performed between module genes and clinical phenotypes. **d** Scatter plot illustrating correlations between Gene Significance (GS) and Module Membership (MM) under the T stage phenotype in the red module genes. Red points represent genes highly associated with the T stage. Statistical test: Pearson’s correlation analysis and two-sided unpaired *t*-test. **e** Scatter plot showing the correlation between GS and MM under the Gleason Score phenotype in the red module genes. Red points represent genes highly associated with the Gleason Score. Statistical test: Pearson’s correlation analysis and two-sided unpaired *t*-test. **f** Scatter plot showing the correlation between GS and MM under the Biochemical Recurrence (BCR) phenotype in the red module genes. Red points represent genes highly associated with BCR. Statistical tests: Pearson’s correlations and two-tailed unpaired *t*-tests. **g** The overlapping RNAs between WGCNA results and all senescence-related genes.

Figure 1 presents this study’s overall design. First, we collected aging-related genes from five publicly available databases (Fig. 2a, Supplementary Data 1). Gene modules linked with clinical features were then identified via co-expression network analysis, with a *β* value of 7 (*R*^2^ = 0.84) considered to be optimum for network construction (Fig. S1A). Overall, 29 distinct modules were identified, each represented by a unique color (Fig. 2b, Fig. S1B) and the eigengene (representing the first principal component of expression in specific modules). Correlations between the gene modules and clinical traits, such as T stage, Gleason score, age, and BCR, were subsequently calculated. Among all module-trait relationships, the red module showed the strongest correlation with clinical traits (Fig. 1c). Additionally, genes were selected as hub prognosis-related genes if they exhibited a module membership (MM) > 0.5 as well as a gene significance (GS) > 0.2 (Fig. 2d–f). Finally, we intersected the 316 genes identified from the WGCNA red module with senescence-related genes from the public databases, resulting in 38 overlapping genes that were considered key candidates for further analysis (Fig. 2g, Supplementary Data 2).

### Construction of the prostate cancer senescence-related gene signature (SRGS)

**Fig. 3:**
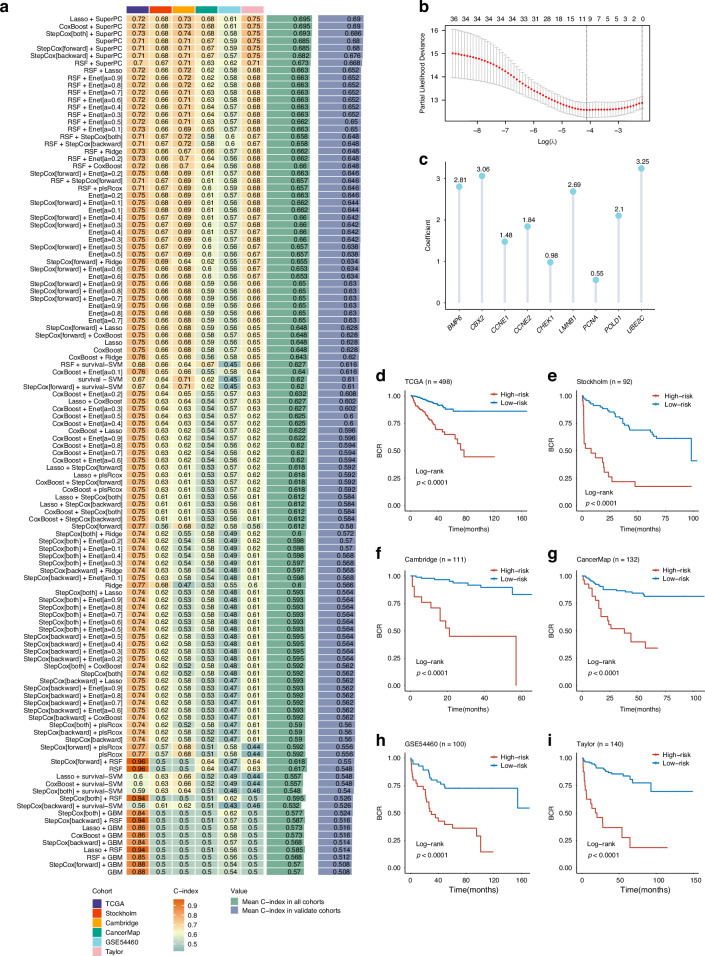
Construction and validation of the prostate cancer senescence-related gene signature (SRGS) via a machine learning-based framework. **a** A total of 117 prediction models were evaluated using the LOOCV framework. The C-index of each model was calculated across all validation cohorts and combined cohorts. **b** The optimal *λ* was calculated from the lowest partial likelihood deviance value in the TCGA-PRAD cohort. **c** Composition and corresponding coefficients of genes included in the SRGS model. **d** Kaplan–Meier curves for Biochemical Recurrence (BCR) according to the SRGS in the TCGA-PRAD cohort (*n* = 498). **e** Kaplan–Meier curves for BCR according to the SRGS in the Stockholm cohort (*n* = 92). **f** Kaplan–Meier curves for BCR according to the SRGS in the Cambridge cohort (*n* = 111). **g** Kaplan–Meier curves for BCR according to the SRGS in the CancerMap cohort (*n* = 132). **h** Kaplan–Meier curves for BCR according to the SRGS in the GSE54460 cohort (*n* = 100). **i** Kaplan–Meier curves for BCR according to the SRGS in the Taylor cohort (*n* = 140).

Based on the 38 genes identified in the previous analysis, a consensus SRGS was constructed using machine-learning integration. Using the TCGA-PRAD cohort, 117 predictive models were trained using the LOOCV framework before calculating the C-index for the individual models in the validation cohorts (Fig. 3a) with the optimal model combining LASSO regression and SuperPC (Fig. 3a, Supplementary Data 3). The optimal λ\lambda value for LASSO regression was determined when the partial likelihood deviance was minimal under the LOOCV framework (Fig. 3b), resulting in a nine-gene signature (Fig. 3c).

After calculating risk score for each patient, we employed the survminer package to determine the cohort-specific optimal cut-off value using BCR as the risk event. This threshold was subsequently used to stratify patients into high- and low-risk groups. Overall, high-risk cases showed markedly worse BCR outcomes relative to those with low-risk in the TCGA-PRAD training dataset and the six independent validation cohorts (all *P* < 0.05) (Fig. 3d–i).

### Evaluation of the SRGS model and comparison with other gene expression-based prognostic signatures

The predictive performance of SRGS was further assessed according to area under the curve (AUC) values for six cohorts: TCGA-PRAD (0.709), Stockholm (0.693), Cambridge (0.68), CancerMap (0.714), GSE54460 (0.6551), and Taylor (0.747) (Fig. S2A). Time-dependent ROC analysis showed AUCs for SRGS at 1, 3, and 5 years of 0.791, 0.738, and 0.664 in TCGA-PRAD; 0.787, 0.756, and 0.7 in Stockholm; 0.83, 0.716, and 0.705 in Cambridge; 0.624, 0.692, and 0.745 in CancerMap; 0.735, 0.66, and 0.634 in GSE54460; and 0.818, 0.773, and 0.783 in Taylor (Fig. S2B).

Additionally, the prognostic significance of SRGS was compared with clinical information across all six cohorts. Notably, SRGS achieved the highest C-index in all cohorts, demonstrating its superior predictive accuracy and stability (Fig. S2C).

In recent years, advances in big-data technologies and next-generation sequencing enabled the application of machine learning for developing various prognostic and predictive gene expression signatures. To benchmark SRGS against existing signatures, we systematically retrieved 60 published signatures (Supplementary Data 4), representing different biological processes, including androgen biosynthesis and catabolism, autophagy, ferroptosis, cuproptosis, immune response, and DNA damage repair. Univariate Cox regression analysis of these signatures across all datasets revealed that SRGS demonstrated significant associations with prognosis (Fig. S3A), underscoring its robustness.

Furthermore, SRGS outperformed the other signatures in terms of C-index across all datasets (Fig. S3B). Unlike many other models, SRGS, reduced to nine genes through LASSO regression and SuperPC, exhibited better extrapolation potential and reduced the risk of overfitting, achieving consistently strong predictive performance across multiple cohorts.

### Validation using an in-house clinical cohort

**Fig. 4:**
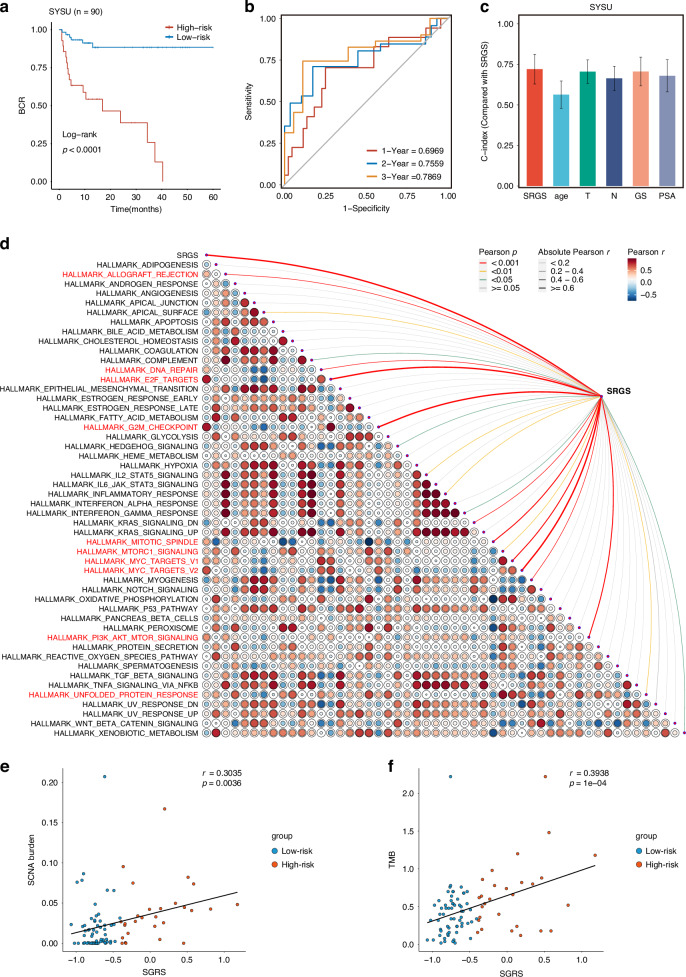
Validation of the SRGS and exploration of its potential mechanisms promoting tumor progression in SYSU-PRAD Cohort. **a** Kaplan–Meier survival curves for Biochemical Recurrence (BCR) according to SRGS in SYSU-PRAD cohort (*n* = 90). **b** Time-dependent ROC analyses for assessing BCR at 1, 2, and 3 years in the SYSU-PRAD cohort. **c** Comparison of SRGS with other clinical factors for prognostic prediction in SYSU-PRAD cohort (*n* = 90). Data represent mean ± 95% CI. **d** Heatmap showing the correlation between SRGS and 50 tumor-associated pathways in SYSU-PRAD cohort. **e** Correlation analysis between SRGS and somatic copy number alterations (SCNA) in SYSU-PRAD cohort. Statistical test: Pearson correlation analysis. **f** Correlation analysis between SRGS and tumor mutational burden (TMB) in SYSU-PRAD cohort. Statistical test: Pearson correlation analysis.

The clinical applicability of the SRGS model was further validated by evaluating its performance in the SYSU-PRAD cohort, comprising RNA sequencing data of 90 PCa patients (Supplementary Data 5). Kaplan–Meier survival analysis indicated significantly worse BCR outcomes for patients with high SRGS scores in comparison with those having low SRGS scores (*P* < 0.0001; Fig. 4a). Time-dependent ROC analysis demonstrated the predictive effectivness. of SRGS, with AUC values of 0.6969, 0.7559, and 0.7869 for predicting BCR at 1, 2, and 3 years, respectively (Fig. 4b).

SRGS was found to be independently predictive of BCR in PCa based on univariate and multivariate Cox analyses (Fig. S4A, B). Furthermore, when comparing the predictive performance of SRGS with other clinical features, SRGS consistently exhibited superior performance (Fig. 4c).

To investigate the biological pathways linked to SRGS, ssGSEA was undertaken to calculate the enrichment scores of 50 hallmark pathway. It revealed a positive correlation between SRGS and pathways related to apoptosis and DNA damage (Fig. 4d), consistent with findings in the TCGA cohort (Fig. S5A). These results align with previous studies suggesting that markers of cellular senescence may promote DNA damage while simultaneously inhibiting apoptosis [5].

As expected, SRGS was positively correlated with both tumor mutational burden (TMB) and somatic copy number alteration (SCNA) in our cohort (*P* < 0.05, Fig. 4e, f). Within the TCGA-PRAD cohort, significantly higher mutation rates in *TP53* and an overall higher TMB were noted for patients in the high SRGS group (Fig. S5B, C). Collectively, these findings suggest that tumors with higher SRGS scores are likely to harbor more extensive genetic alterations, potentially driving further tumor progression.

### SRGS promotes prostate cancer disease progression

**Fig. 5:**
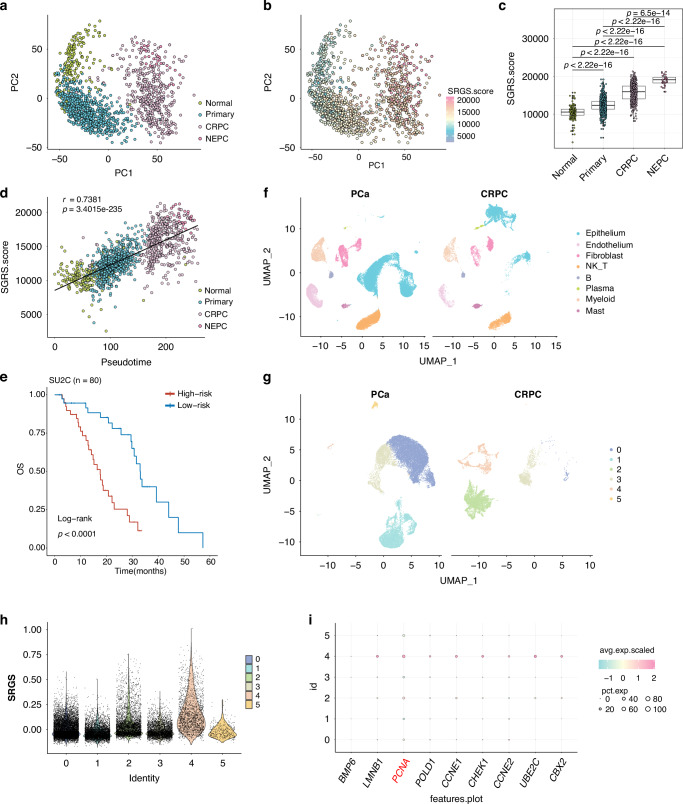
Potential role of SRGS in the progression of prostate cancer. **a** PCA of pan-prostate cancer transcriptomes from normal prostate (green), primary prostate cancer (blue), castration-resistant prostate cancer (CRPC, purple), and neuroendocrine prostate cancer (NEPC, dark red). **b** PCA visualizing SRGS activation during prostate cancer disease progression. **c** Boxplots showing SRGS scores across different prostate cancer progression stages. Statistical test: two-sided unpaired Wilcoxon test. **d** Correlation analysis between SRGS scores and pseudotime. Statistical test: Pearson correlation analysis. **e** Kaplan–Meier survival curves for overall survival (OS) according to SRGS in SU2C cohort (*n* = 80). **f** UMAP visualization of the tumor microenvironments in primary prostate cancer and CRPC; different colors represent different cell types. **g** UMAP visualization of epithelium subsets in primary prostate cancer and CRPC; different colors represent different clusters. **h** Violin plots showing SRGS scores across different epithelial cell clusters. **i** Dot plot showing SRGS gene set levels in different epithelial cell clusters.

Given the potential of SRGS to promote PCa progression, we analyzed RNA-seq profiles from over 1000 human prostate samples to characterize its dynamic role across different disease stages. Patients were first stratified based on their progression from normal epithelial tissue to primary tumor, and subsequent CRPC, and neuroendocrine (NE) PCa, using a previously published tool [25] (Fig. 5a). Notably, we observed that SRGS was significantly upregulated throughout PCa progression (Fig. 5b, c), and positively correlated with bulk tumor pseudotime analysis (*P* < 0.0001, Fig. 5d). Importantly, we found that the SRGS also effectively predicted overall survival in patients with CRPC (Fig. 5e). These results highlight the potential utility and clinical relevance of SRGS in advanced PC and CRPC.

Since bulk RNA-seq data does not provide detailed information on the tumor cell expression profiles, single-cell RNA sequencing was conducted on data from five in-house primary PCa patients and five CRPC patients from public databases [26]. After annotation, we identified nine distinct cell subpopulations. NK cells and T cells were combined into the NK_T cell group, resulting in a total of eight subpopulations: Epithelium, Endothelial, Fibroblast, NK_T, B, Plasma, Myeloid, and Mast cells. Tumor epithelial cells represented the largest proportion of the single-cell population (Fig. S6A–C). Although epithelial cells remained the predominant cell type across different disease stages, we observed significant heterogeneity within tumor epithelial cells (Fig. 5f, g, Fig. S6D), which is consistent with prior study [27].

We next evaluated the expression of SRGS in epithelial cell subpopulations and found that clusters 2 and 4 exhibited higher SRGS expression (Fig. 5h). Notably, clusters 2 and 4 were more prevalent in CRPC. Additionally, we observed that epithelial cluster 4 was significantly enriched in metabolism-related pathways, while epithelial cluster 2 showed increased expression of ribosomal pathways (Fig. S7A). The results align with previous ones suggesting that subpopulations with high expression of aging-related genes, despite being in a relatively quiescent G1 phase, are still metabolically active [28]. This metabolic state may serve as a protective mechanism, promoting further tumor progression.

Further analysis with inferCNV revealed that clusters 2 and 4 had significantly higher copy number variations (CNVs) compared to other clusters (Fig. S7B, C). This finding supports the idea that tumor cells with high SRGS expression may harbor greater genetic instability, potentially driving tumor progression (Fig. 4e, f). Interestingly, when the expression of individual genes within the SRGS was examined, we found that *PCNA* was highly expressed in clusters 2 and 4 (Fig. 5i), indicating that *PCNA* could be involved in promoting PCa progression.

### *PCNA* as a potential therapeutic target for high-risk PCa

**Fig. 6:**
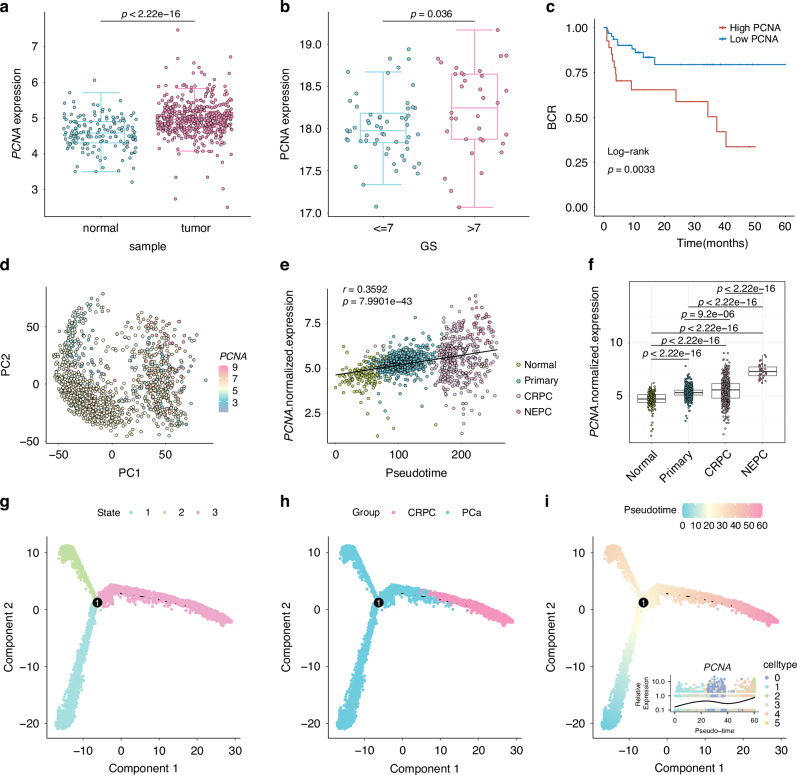
PCNA as a target for treating high-risk prostate cancer. **a** Boxplots comparing PCNA expression between cancerous and normal prostate prostate tissues in the TCGA and GTX datasets. Statistical test: two-sided unpaired Wilcoxon test. **b** Boxplots showing PCNA protein levels in Gleason-score >7 vs Gleason-score ≤7 groups in the SYSU-PRAD protein cohort (*n* = 90). Statistical method: two-sided unpaired Wilcoxon test. **c** Kaplan–Meier survival curves of biochemical recurrence (BCR) stratified by PCNA protein expression in the SYSU-PRAD protein cohort (*n* = 90). **d** Visualization of PCNA activation across prostate cancer disease progression stages. **e** Scatter plot analyzing the correlation between PCNA expression and prostate cancer disease progression using Pearson correlation analysis. **f** Boxplots illustrating PCNA expression across different prostate cancer stages. Statistical method: two-sided unpaired Wilcoxon test. **g**–**i** Pseudotime analysis of cell trajectories utilizing Monocle 2 to assess epithelial cellular dynamics during prostate cancer progression: **g** Visualization in state. **h** Visualization in different group. **i** Visualization of pseudotime progression with changes in PCNA expression.

PCNA’s role in promoting PCa was investigated by analyzing *PCNA* expression in TCGA and GTX datasets. It was found that *PCNA* levels were significantly higher in PCa tissues in comparison with normal ones (Fig. 6a). We further validated this finding in the SYSU-PRAD cohort, comprising 90 PCa patients, using protein expression data. In patients with a gleason score >7, PCNA protein expression was notably elevated (Fig. 6b). Moreover, high PCNA expression correlated with poorer BCR outcomes (Fig. 6c).

Interestingly, we also observed significant upregulation of *PCNA* during PCa progression, with its expression being positively correlated with pseudotime analysis of bulk tumor progression (*P* < 0.0001, Fig. 6d–f). Additionally, single-cell monocle analysis revealed two distinct ways in tumor epithelial cells, one of which progressed toward CRPC. During this progression, *PCNA* expression gradually increased, whereas the other SRGS genes showed relatively low contribution (Fig. 6g–i, Fig. S8A–C). These findings further support *PCNA*’s role in promoting PCa progression and present it as a promising target for therapy.

### *PCNA* as a therapeutic target for high-risk PCa

**Fig. 7:**
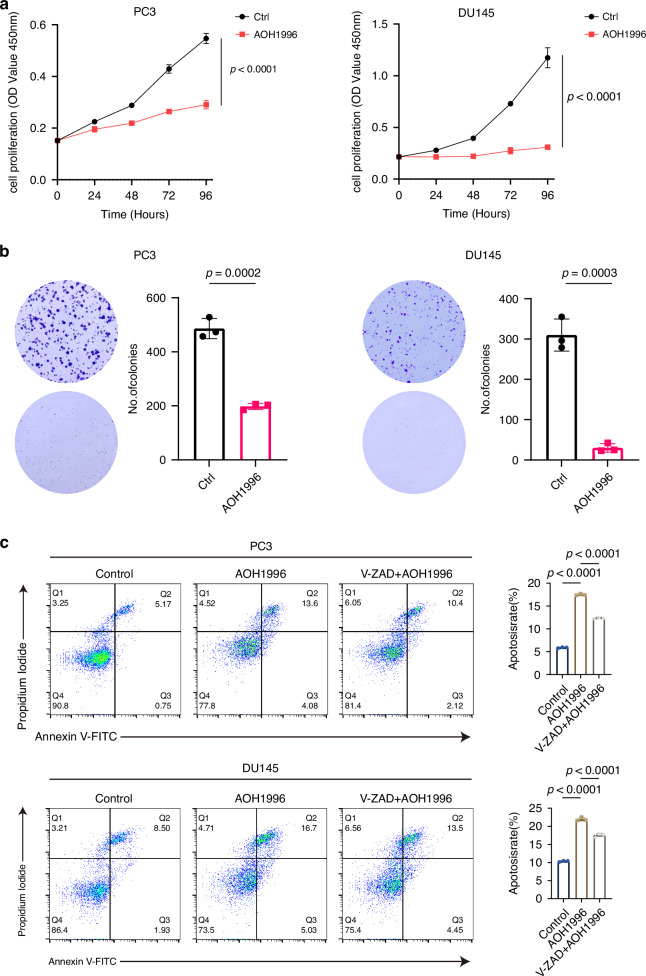
In vitro validation of PCNA as a therapeutic target for prostate cancer. **a** CCK-8 assay demonstrating the effect of AOH1996 on the proliferation of PC3 and DU145 cells. Statistical method: two-sided unpaired *t* test. **b** Colony formation assays showing the influence of AOH1996 on the clonogenicity of PC3 and DU145 cells. Statistical method: two-sided unpaired *t* test. **c** Flow cytometry apoptosis analysis revealing the effect of AOH1996 on the proportions of apoptotic PC3 and DU145 cells. Statistical method: one way ANOVA followed by Dunnett’s test.

We inhibited *PCNA* with PCNA inhibitor AOH1996 in PC3 and DU145 cells to further validate its role in PCa. We confirmed the expression of PCNA in PC3 and DU145 cells using Western blotting and qPCR (Fig. S9A, B). In addition, consistent results were observed in the CTPC dataset and in our previously published study [29, 30] (Fig. S9C, D). Given the previously suggested involvement of PCNA in regulating cell proliferation and apoptosis, we performed CCK-8 and colony formation assays. It was found that treatment with AOH1996 markedly reduced the proliferation of PC3 and DU145 cells (Fig. 7a, b). Additionally, apoptosis assays revealed that PCNA inhibition significantly increased the proportion of apoptotic PC3 and DU145 cells, while treatment with Z-VAD-FMK, a pan-caspase inhibitor, effectively reduced the proportion of apoptotic cells. (Fig. 7c).

**Fig. 8:**
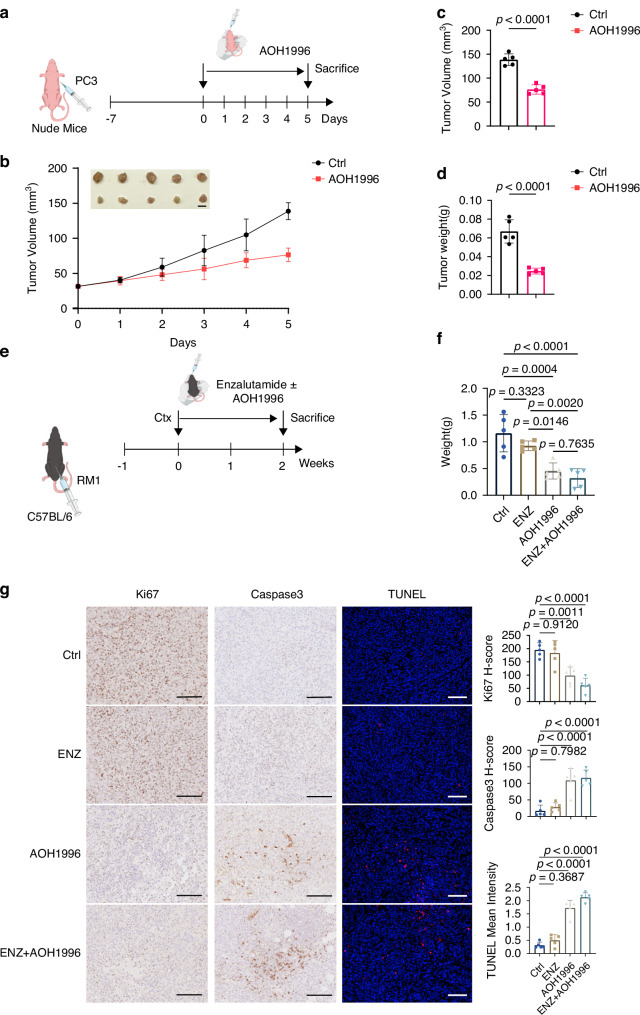
In vivo validation of PCNA inhibition in suppressing prostate cancer tumor growth. **a** Experimental scheme for PC3 subcutaneous xenografts in nude mice. **b** Representative images of tumors and volume for 6 days in subcutaneous xenograft mice. The scale bar presents 5 mm. **c**, **d** Statistical analysis of tumor volume and tumor weight in different groups of nude mice. **e** Experimental scheme for RM1 orthotopic xenografts in C57BL/6 mice. **f** Statistical analysis of tumor weight in different groups of RM1 orthotopic xenografts. Data are shown as mean and SD. Statistical method: one-way ANOVA with Dunnett’s test. **g** Representative images of Ki67 immunohistochemical staining, Caspase3 immunohistochemical staining, and TUNEL staining. The scale bar presents 100 μm. Data are shown as mean and SD. Statistical method: one-way ANOVA with Dunnett’s test.

The role of *PCNA* in vivo was also validated by inducing subcutaneous tumors in nude mice (Fig. 8a). Consistent with the in vitro findings, tumors treated with PCNA inhibitor exhibited markedly slower growth relative to the controls. On day 5, tumors were harvested, with analysis revealing a marked decrease in both tumor volume and weight for the PCNA-inhibited groups in comparison with the control (Fig. 8b–d).

To better mimic the PCa microenvironment, we established orthotopic tumor models in 4-week-old C57BL/6 mice (Fig. 8e). After treatment with AOH1996, tumor weight measurements demonstrated that orthotopic tumor growth was significantly attenuated. Notably, the combination of AOH1996 with enzalutamide further enhanced this effect (Fig. 8f). IHC staining revealed increased caspase-3 expression but reduced Ki67 expression in tumors following treatment with the PCNA inhibitor alone or in combination with enzalutamide. TUNEL assay results further demonstrated that treatment with the PCNA inhibitor alone or in combination with enzalutamide, significantly increased the proportion of apoptotic tumor cells (Fig. 8g).

Altogether, the findings confirmed that PCNA inhibitors can lower resistance to ADT while enhancing enzalutamide’s efficacy in treating PCa.

### Ethics approval

All animal experiments complied with the ethics of animal experiments in the Experimental Animal Center of Sun Yat-sen University ([2025] no. 011).

## Discussion

Cellular senescence plays a pivotal role in cancer pathogenesis. While early-stage senescence can suppress tumor growth, tumor cells often acquire mechanisms to evade immune surveillance and develop treatment resistance at later stages [31]. In PCa, a malignancy with slow progression, the shift from a senescent state to a proliferative one may drive tumor progression [6, 32]. To identify this transition, we developed a SRGS that effectively stratifies high-risk PCa patients and predicts BCR, a critical clinical event that markedly accelerates disease progression [10]. Through temporal trajectory analysis of bulk RNA-seq data, we found that SRGS, an independent prognostic factor, was significantly associated with ADT resistance. This unveils a previously unexplored biological relevance of senescence in PCa progression that earlier models based solely on senescence-related genes failed to capture. Thus, unlike other gene signatures, SRGS may function not only as a prognostic indicator but also as a valuable tool for the early identification of patients at risk for ADT resistance and disease progression. Furthermore, SRGS demonstrated significant predictive value for clinical outcomes in patients received second-generation androgen receptor inhibitors.

Although ADT remains the first-line therapy for advanced PCa, its efficacy diminishes over time due to androgen receptor (AR) reactivation and disruption of proliferative signaling pathways [33]. Furthermore, patients with CRPC often exhibit poor tolerance to chemotherapies like docetaxel, due to severe adverse effects [34]. To further investigate potential therapeutic targets in high-risk and CRPC populations, we examined the biological implications of SRGS and observed its strong association with genomic instability and DNA damage repair pathways. This suggests that SRGS may mark the senescence-to-proliferation transition, contributing to genetic alterations and malignant progression.

The nine genes constituting the SRGS include those closely associated with cell cycle regulation and proliferation (CCNE1, CCNE2, CHEK1, BMP6, CBX2, LMNB1, and UBE2C), as well as genes involved in DNA damage repair (PCNA and POLD2), all of which are functionally linked to the process of senescence progression [35]. Notably, BMP6 has been reported to be markedly upregulated during senescence and to play a crucial role in advanced PCa and CRPC bone metastasis [36, 37]. CBX2 and CHEK1 have been implicated in the development of NEPC [38, 39]. Furthermore, genes related to proliferation and DNA repair have been previously demonstrated to contribute to PCa initiation, progression, and transition to CRPC [40–43]. These molecular characteristics may underlie the ability of the SRGS to predict overall survival in patients with CRPC.

Moreover, through temporal trajectory analyses of both single-cell and bulk transcriptomic data, PCNA was identified among SRGS as a key driver of PCa progression. PCNA is a critical regulator of the cell cycle, cellular proliferation, and apoptosis [44, 45]. Elevated PCNA expression has been observed across various cancers and is often correlated with poor clinical outcomes [46]. Our findings indicate that high PCNA expression may enable senescent tumor cells to escape growth arrest and re-enter the cell cycle, driving tumor progression.

Loss or mutation of p53, which is a common alteration in patients with high SRGS scores and a frequent event during PCa progression, may also drive the transition from hormone-sensitive PCa (HSPC) to castration-resistant PCa (CRPC) [47]. Previous research has demonstrated that p53 regulates PCNA to maintain genomic stability during DNA replication [48, 49]. Our results also support a potential biological mechanism by which targeting PCNA may enhance the efficacy of other DNA-damaging agents in the treatment of PCa [50]. In addition, it has shown that PCNA regulated by FOXM1, can promote PCa cell proliferation through a p53-independent mechanism [51]. We observed that elevated PCNA levels were associated with increased mutational burden and enhanced cell proliferation, further supporting its role in disease advancement. Furthermore, it has been reported that Y211 phosphorylation of PCNA is a frequent event in advanced PCa [52]. These findings suggest that early detection of PCNA overexpression could aid in identifying patients at higher risk for progression.

Recent studies have introduced AOH1996, a novel small-molecule inhibitor that selectively targets PCNA and has shown significant efficacy in various tumor cells [53]. AOH1996 promotes the interaction between PCNA and RPB1, disrupts PCNA binding to active chromatin, and induces transcription-dependent DNA double-strand breaks. However, it is worth noting that establishing the structural basis for targeting such a highly flexible and dynamic protein–protein interface remains a major challenge. Therefore, future studies are needed to further investigate the potential adverse effects of AOH1996, including possible hepatic and renal toxicities. Notably, AOH1996 has been shown to exhibit low toxicity both in vitro and in vivo, and has been approved by the FDA to enter Phase I clinical trials in the United States for the treatment of refractory solid tumors [54]. In our study, AOH1996 effectively inhibited PCa cell proliferation and induced apoptosis. In vivo experiments further validated its capacity to suppress tumor growth. Importantly, combining AOH1996 with the AR inhibitor enzalutamide produced synergistic effects, highlighting the potential of this combination strategy in clinical settings. Future studies should explore the integration of PCNA inhibitors like AOH1996 with existing treatments, including immunotherapy, chemotherapy, and radiotherapy, to improve clinical outcomes and quality of life for PCa patients.

In conclusion, we developed a robust SRGS using 117 machine learning methods, enabling accurate prognostication and identification of patients at risk for disease progression. Our findings emphasize the translational potential of SRGS in clinical decision-making and underscore PCNA as a promising therapeutic target, especially in high-risk and CRPC patients.

## Supplementary information

Supplementary information

Supplementary Data 1

Supplementary Data 2

Supplementary Data 3

Supplementary Data 4

Supplementary Data 5

## Data Availability

The sources for all public datasets utilized in this study are provided in Supplementary Table. The SYSU-PRAD cohort data has been deposited in https://ngdc.cncb.ac.cn/gsa-human/browse/HRA008293.

## Code availability

This study did not produce any new analytical code. All computational procedures have been fully described in the “Methods” section. Additional details can be obtained from the corresponding author upon reasonable request.
