# Supplementary material for: Senescence-related gene signature predicts prostate cancer progression and identifies PCNA as a therapeutic target via multi-omics machine learning integration
Source: Br J Cancer. 2025 Dec 18;134(4):662–75. doi: 10.1038/s41416-025-03309-6 (PMC12858972; doi:10.1038/s41416-025-03309-6)
Supplement: Supplementary file 1 — Supplementary information [file 41416_2025_3309_MOESM1_ESM.docx]

**Supplementary Information**

**Senescence-related gene signature predicts prostate cancer progression and identifies PCNA as a therapeutic target via multi-omics machine learning integration**

Renxuan Lin *et al.*

*Corresponding author: Zong-Ren Wang, wangzr27@mail.sysu.edu.cn; Ren Liu, liur227@mail.sysu.edu.cn.

**Supplementary Table 1. Reagents and resources.**

| **Reagent or Resource** | **Source** | **Identifier** |
| --- | --- | --- |
| **Cell lines** | | |
| RM-1 | iCell | iCell-m051 |
| PC3 | iCell | iCell-h174 |
| DU145 | iCell | iCell-h250 |
| **Antibodies** | | |
| PCNA(PC10) Mouse mAb #2586 | CST | Cat# 2586T |
| Rabbit monoclonal [SP6] to Ki67 | Abcam | Cat# ab16667 |
| **Chemicals** |  |  |
| AOH1996 | Abmole | Cat# M40519 |
| Enzalutamide | MCE | Cat# HY-70002 |
| Z-VAD-FMK | MCE | Cat# HY-16658B |
| Deposited data |  |  |
| TCGA-PRAD |  |  |
| Stockholm |  | GSE70769 |
| Cambridge |  | GSE70768 |
| CancerMap |  | GSE94767 |
| GSE54460 |  | GSE54460 |
| Taylor |  | GSE21034 |
| GTEX |  | https://xenabrowser.net/ |
| Bulk-RNAseq human prostate cancer | Bolis et al. | https://prostatecanceratlas.org |
| SU2C |  | cBioPortal |
| CTPC |  | https://pcatools.shinyapps.io/CTPC_V2/ |
| CRPC scRNA-seq |  | GSE137829 |

**Supplementary Table 2. List of primers**

| Gene | Forward primer (5’- 3’) | Reverse primer (5’- 3’) |
| --- | --- | --- |
| PCNA | CCTGCTGGGATATTAGCTCCA | CAGCGGTAGGTGTCGAAGC |
| ACTB | CATGTACGTTGCTATCCAGGC | CTCCTTAATGTCACGCACGAT |


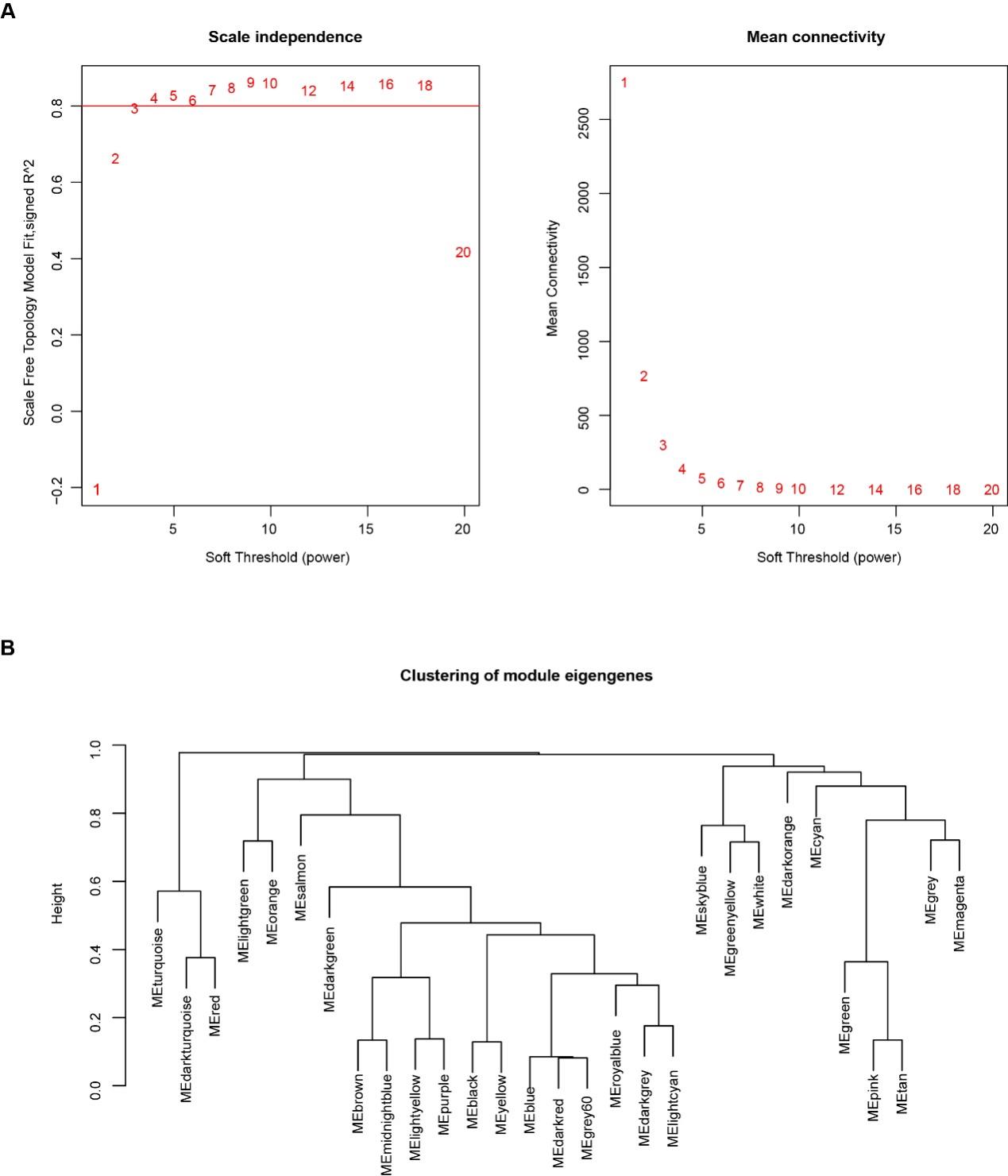


**Figure S1. Construction of WGCNA in TCGA-PRAD**

**A.** Network topology analysis for different soft-threshold powers. The effects of soft-threshold power on the topology-fit index (scale-free) are shown in the left-hand panel.

**B.** Clustering Dendrogram of Module Genes.


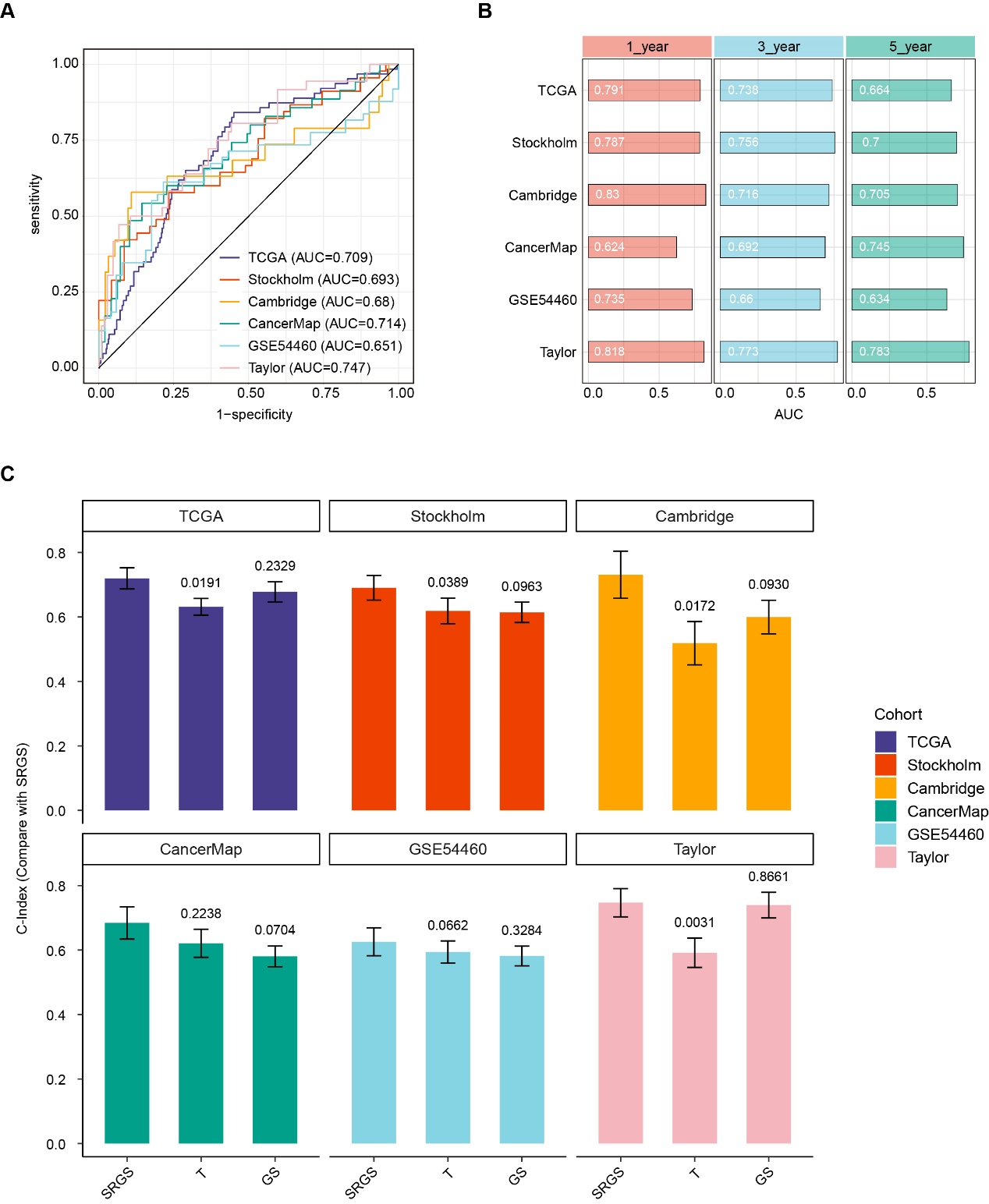


**Figure S2. Assessment of the SRGS model**

**A.** Receiver Operating Characteristic (ROC) curves for SRGS predicting biochemical recurrence (BCR) across all cohorts, with the corresponding area under the curve (AUC) values.

**B.** Time-dependent ROC analysis for predicting Biochemical Recurrence (BCR) at 1, 3, and 5 years across all cohorts.

**C.** Comparison of the performance of SRGS with those of other clinical factors in predicting prognosis. Statistical tests: two-sided z-score test. Data are shown as mean ± 95% CI.


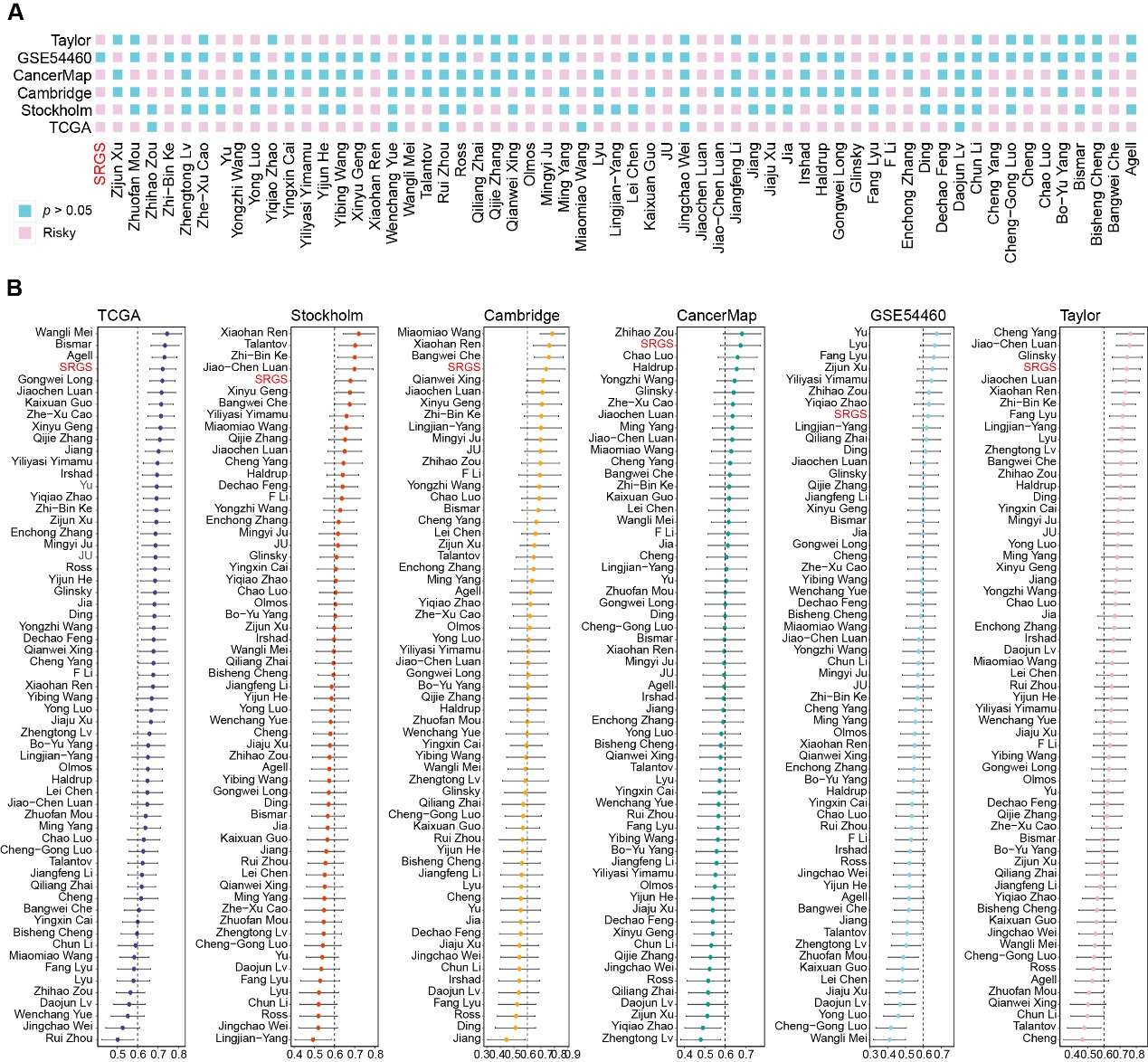


**Figure S3. Comparison of prognostic models in prostate cancer cohorts**

**A.** Univariate Cox regression analysis of SRGS and 60 published gene signatures in TCGA-PRAD, Stockholm, Cambridge, CancerMap, GSE54460, and Taylor.

**B.** C-index analysis comparing SRGS with 60 published gene signatures in TCGA-PRAD, Stockholm, Cambridge, CancerMap, GSE54460, Taylor. Data are presented as mean ± 95% CI.


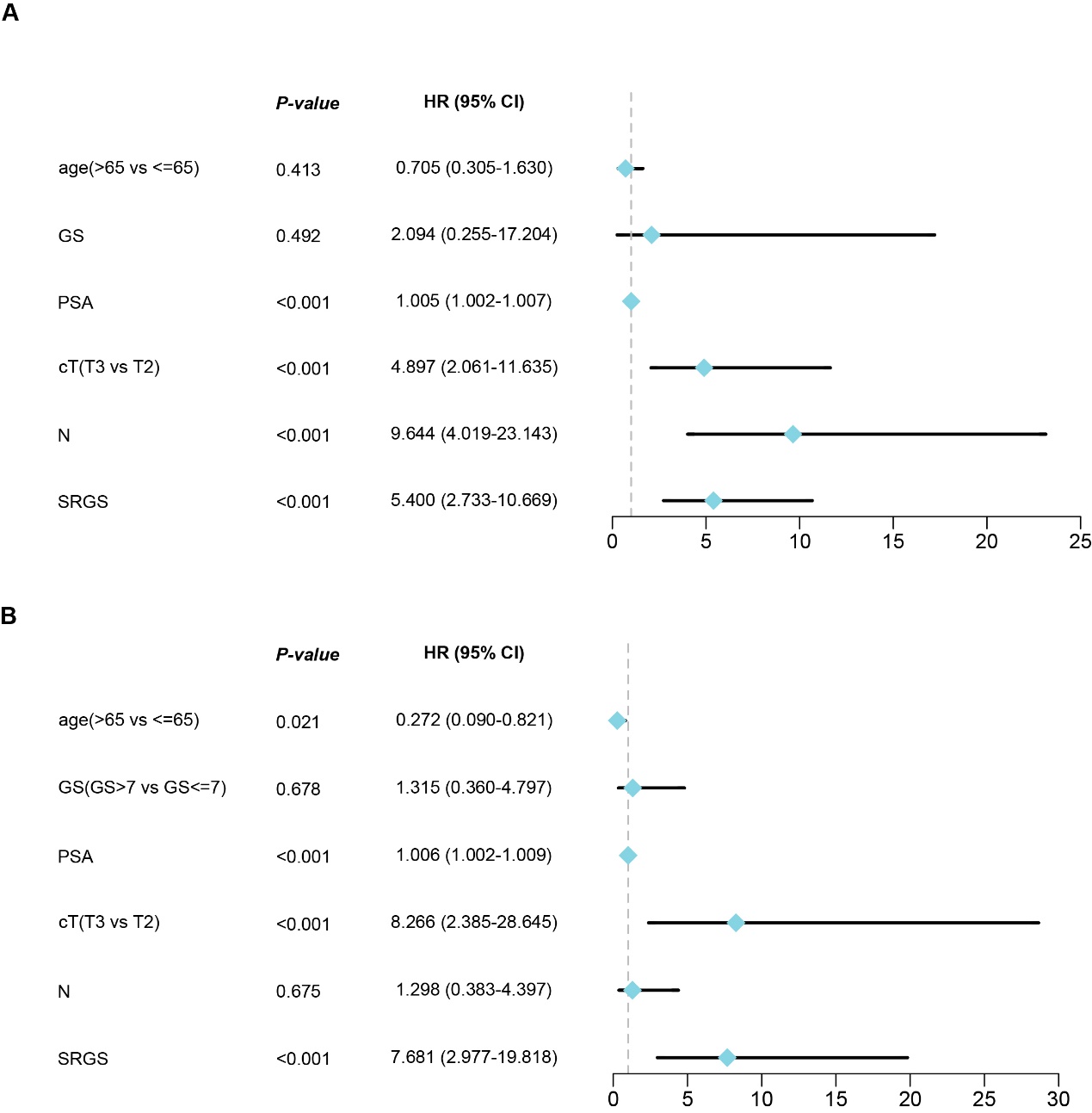


**Figure S4. Validation of SRGS as an independent risk factor in SYSU-PRAD cohort**

**A.** Results of the univariate Cox regression analysis in the SYSU-PRAD cohort (n = 90).

**B.** Results of the multivariate Cox regression analysis in the SYSU-PRAD cohort (n = 90).


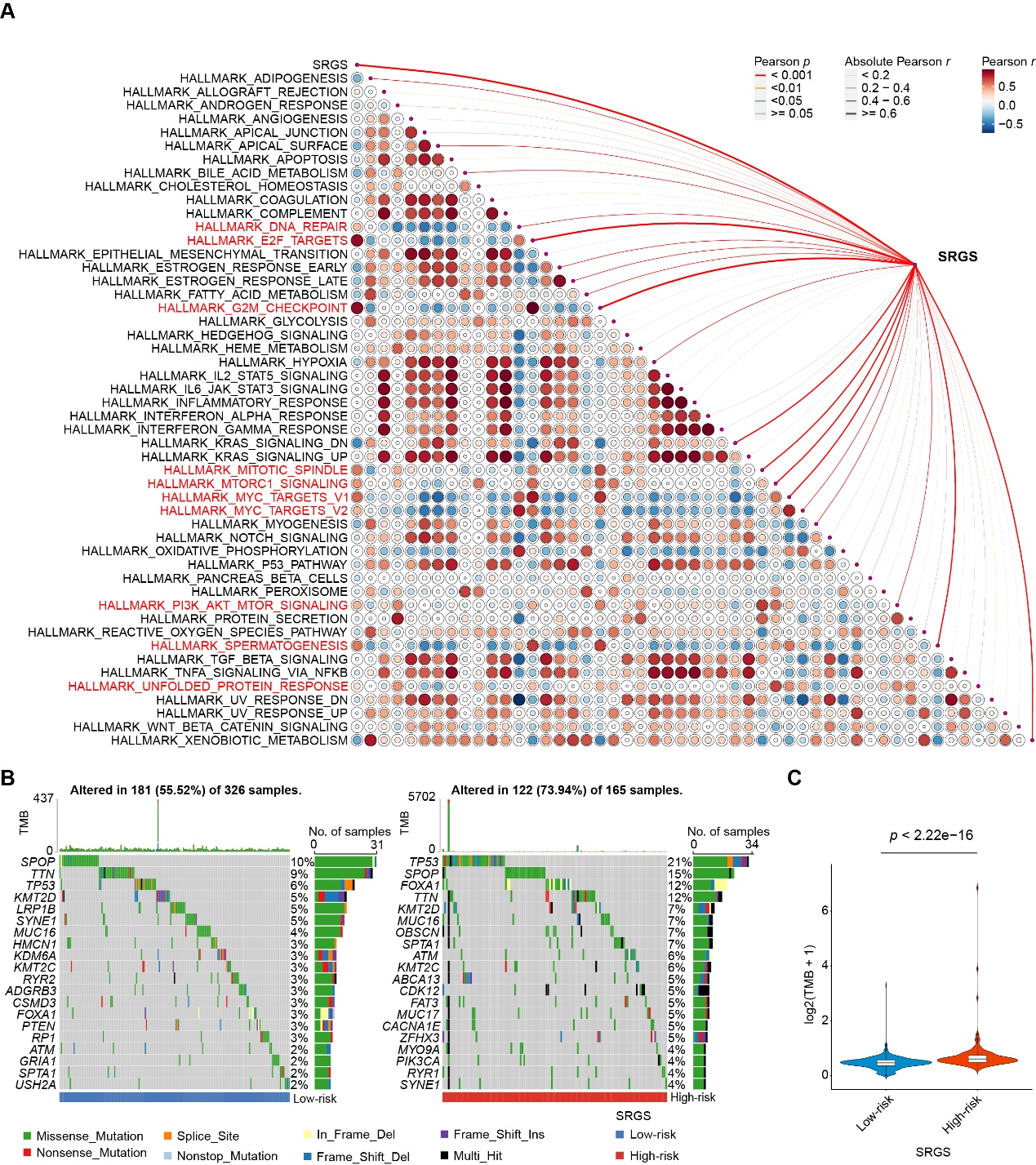


**Figure S5.** **Exploration of potential mechanisms promoting tumor progression in TCGA-PRAD Cohort**

**A.** Heatmap showing correlations between SRGS and 50 tumor-associated pathways in TCGA-PRAD cohort.

**B.** Genomic mutation profiles in TCGA-PRAD cohort based on SRGS group. Tumor mutational burden (TMB) in each tumor sample (top). Frequently mutated prostate cancer-associated genes and their occurrence in tumor samples (bottom). Mutation frequencies are represented by a bar plot.

**C.** Comparison of the overall TMB in TCGA-PRAD cohort based on SRGS group. Statistical test: two-sided unpaired Wilcoxon test.


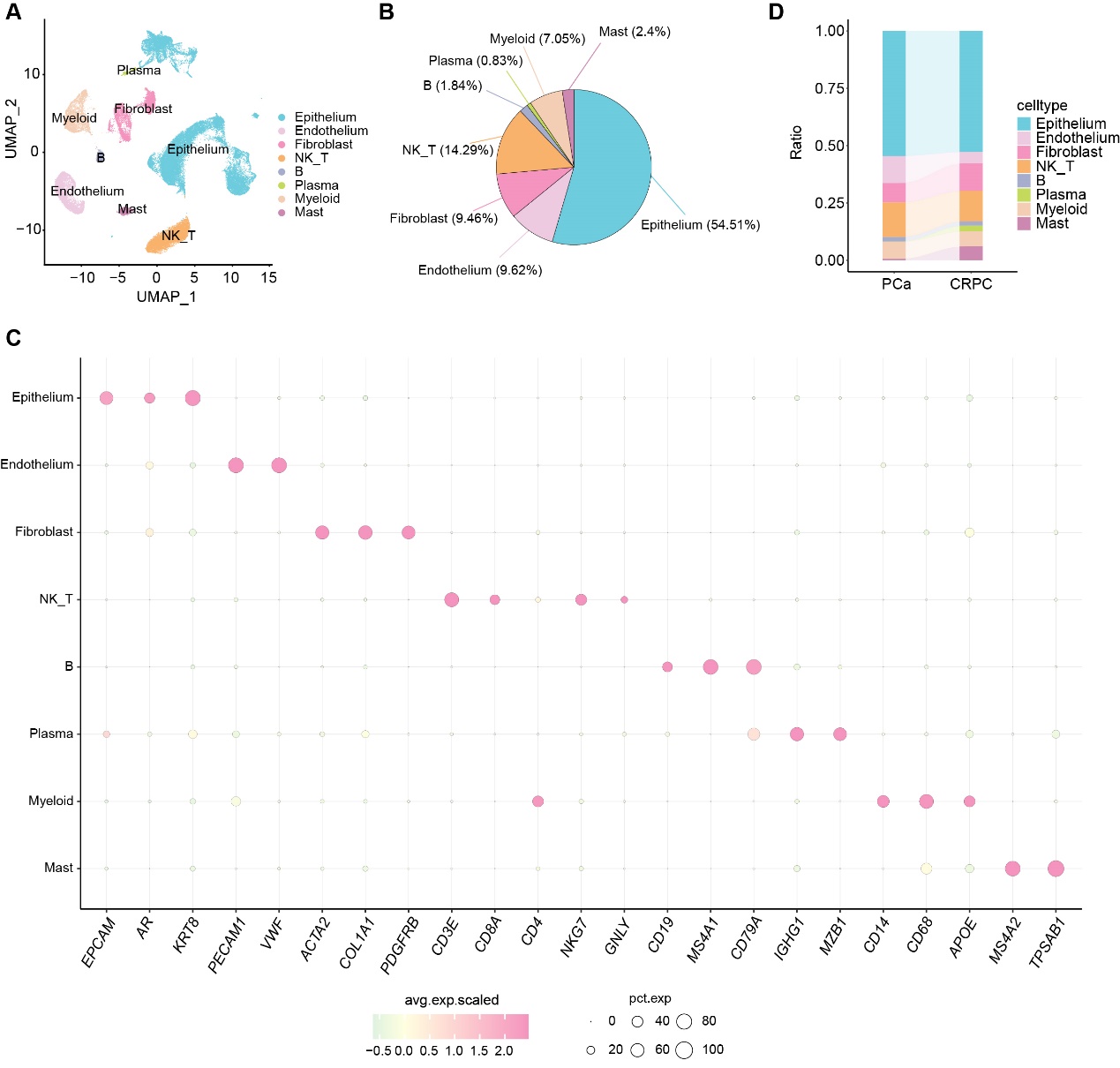


**Figure S6. Single cell analysis of the prostate cancer tumor microenvironment**

**A.** UMAP visualization of the prostate cancer tumor microenvironment, where each color represents a specific cell type.

**B.** Pie chart showing the proportions of different cellular types in prostate cancer.

**C.** Dot plot showing marker gene levels in various cell types in prostate cancer.

**D.** Stacked bar plots showing the composition of cell proportions in primary prostate cancer and CRPC.


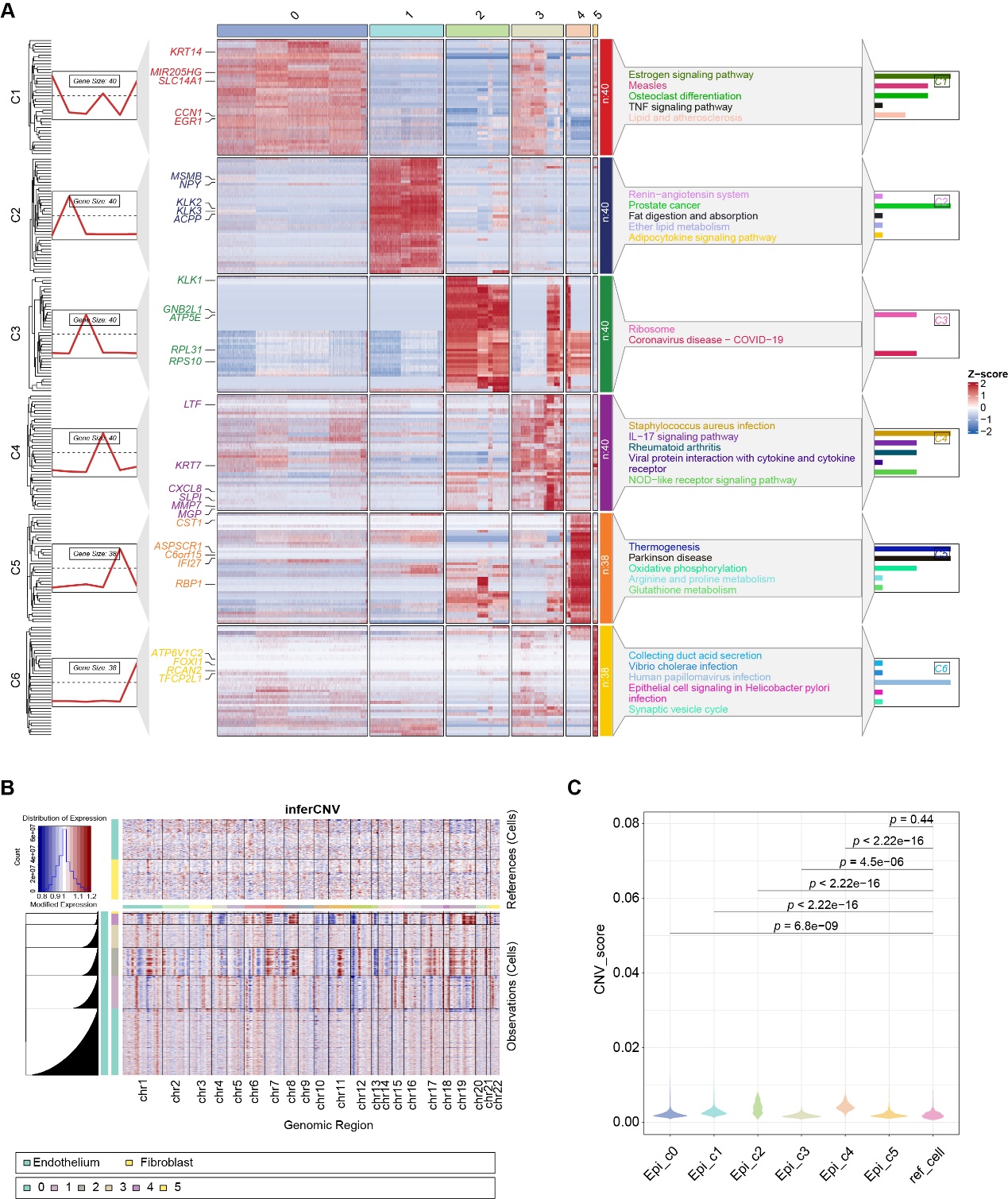


**Figure S7. Analysis of epithelium subsets in prostate cancer**

**A.** Heatmap showing the differential genes of various epithelial cell clusters in prostate cancer, with the top five enriched pathways for these differential genes.

**B.** CNV heatmap visualized using inferCNV analysis, highlighting the copy number variations across different epithelial cell clusters in prostate cancer.

**C.** CNV scores between epithelial cell subclusters and reference cells. Statistical test: two-sided unpaired Wilcoxon test.


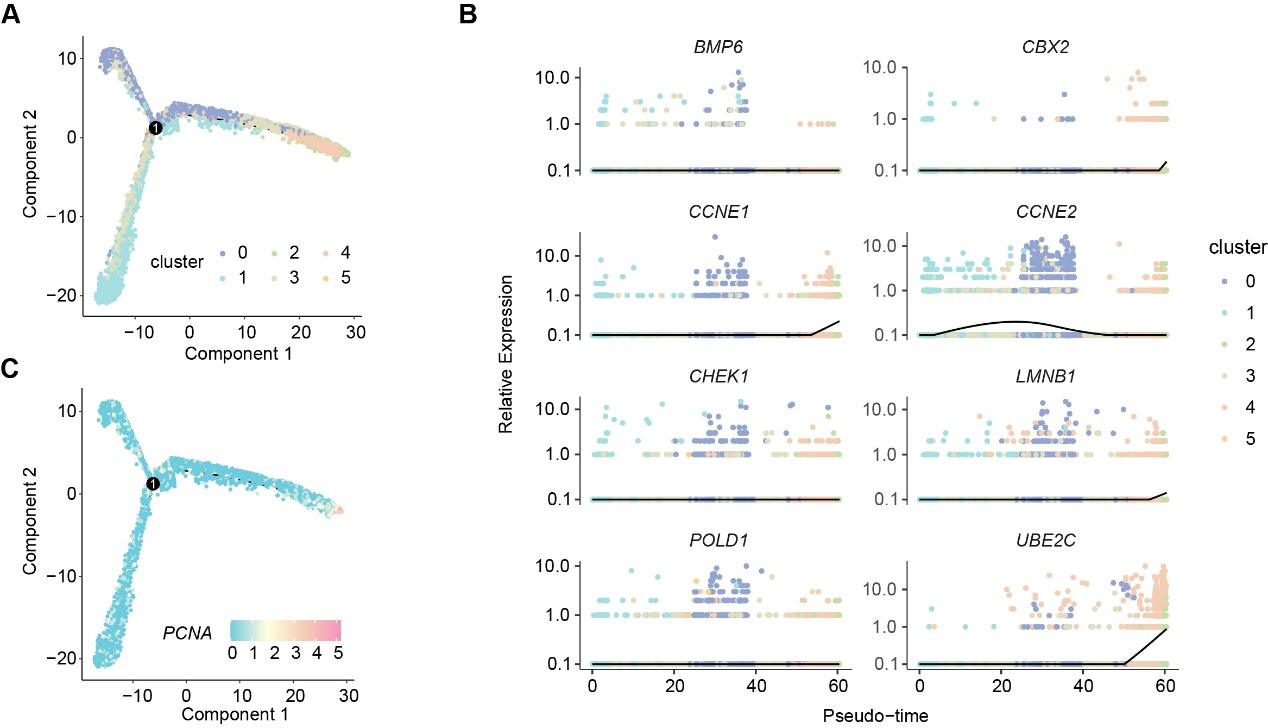


**Figure S8. Pseudotime analysis of epithelium subsets in prostate cancer**

**A.** Pseudotime cell trajectories analysis using Monocle 2, visualizing the dynamic changes within epithelial clusters during prostate cancer progression.

**B.** Expression changes of eight genes in SRGS during pseudotime progression.

**C.** The expression dynamics of PCNA along the pseudotime trajectory.


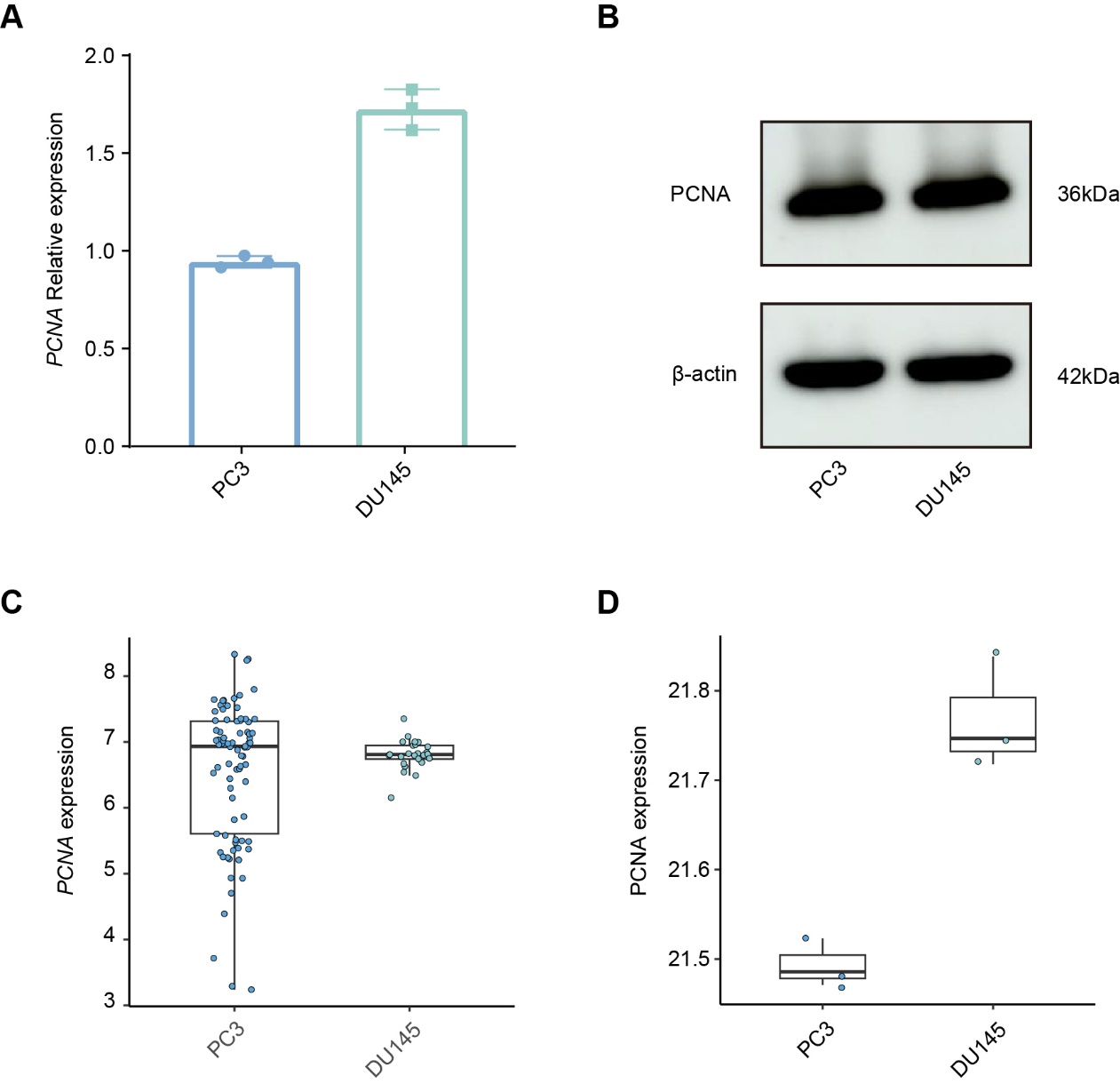


**Figure S9. Expression levels of PCNA in PC3 and DU145 cells.**

**A.** PCNA mRNA expression levels in PC3 and DU145 cells determined by RT–qPCR.

**B.** Protein expression levels of PCNA in PC3 and DU145 cells assessed by Western blot.

**C.** PCNA mRNA expression levels of PC3 and DU145 cells in the CTPC dataset.

**D.** PCNA protein expression levels in PC3 and DU145 cells based on the proteomic data reported by *Ou et al*.
